# Examining the role of emotion regulation, anger, and anxiety in misophonia: A network model

**DOI:** 10.1371/journal.pone.0329920

**Published:** 2025-08-18

**Authors:** Matthew R. Hanna, Amanda C. Collins, Yanyan Shan, Bill Chen, Siyuan Wang, M. Zachary Rosenthal

**Affiliations:** 1 Department of Psychology & Neuroscience, Duke University, Durham, North Carolina, United States of America; 2 Center for Technology and Behavioral Health, Geisel School of Medicine, Dartmouth College, Lebanon, New Hampshire, United States of America; 3 Department of Biomedical Data Science, Geisel School of Medicine, Dartmouth College, Lebanon, New Hampshire, United States of America; 4 Department of Biomedical Engineering, Duke University, Durham, North Carolina, United States of America; 5 Department of Psychiatry & Behavioral Sciences, Duke University School of Medicine, Durham, North Carolina United States of America; Chulalongkorn University Faculty of Psychology, THAILAND

## Abstract

**Background:**

Misophonia, characterized by intense negative reactions to specific sounds, is associated with significant emotional distress. The connections among misophonia severity and factors like emotion regulation, anxiety, and anger remain unclear. This study uses network analysis to clarify these relationships in adults with self-reported misophonia symptoms, identifying key intervention targets and processes driving symptom severity.

**Methods:**

A community sample of adults with misophonia symptoms and impairment (*N* = 205) completed psychometrically validated self-report measures, including the Duke Misophonia Questionnaire (DMQ), Misophonia Questionnaire (MQ), and assessments of emotion regulation, anxiety, and anger. Network analysis was conducted to identify associations among misophonia severity, anxiety, anger, and emotion regulation components. Centrality indices were used to evaluate the most influential factors in the network, and community detection was employed to explore underlying clusters.

**Results:**

Misophonia severity was most strongly associated with emotional awareness, nonacceptance, anxiety, and anger. The network analysis revealed that nodes representing emotion regulation strategies, nonacceptance, and impulsivity had the highest centrality and expected influence values, indicating their significant role in the overall network. Community detection identified two distinct clusters: one reflecting emotion dysregulation and misophonia, and the other related to emotional clarity and awareness.

**Conclusions:**

This study highlights the importance of nonacceptance, emotional awareness, anger, and anxiety in understanding misophonia severity. Interventions targeting anger, anxiety, and nonacceptance may be most effective in managing misophonia symptoms. Future research should explore these relationships longitudinally to better inform treatment approaches.

## Introduction

Misophonia is a recently recognized disorder characterized by strong, negative emotional reactions to specific auditory stimuli, often referred to as “trigger sounds” [1–3]. These sounds are typically human-generated, such as chewing, sniffing, or throat clearing, and can provoke intense emotional responses that others might not commonly experience [1,2,4–7]. Individuals with misophonia experience significant distress and impairment in various aspects of daily life, including social, occupational, and personal domains [1,5,8–10]. Although misophonia is not currently classified as a distinct disorder in any disciplinary nomenclature, it is increasingly recognized as a serious condition with a unique constellation of symptoms, warranting further investigation into its mechanisms and treatment approaches.

The prevalence of clinically significant misophonia is estimated in the population to be between 2–5% [11–13]. This prevalence underscores the critical need for greater awareness, understanding, and research into the condition to better manage its debilitating effects. Notably, the prevalence of misophonia is comparable to or exceeds that of several other psychiatric disorders. For instance, generalized anxiety disorder, borderline personality disorder, obsessive-compulsive disorder, autism spectrum disorder, and schizophrenia have well-documented prevalence rates that are lower than or similar to estimates of misophonia prevalence [14–18].

Understanding misophonia requires exploring its underlying mechanisms, which involve neurological, physiological, and psychological factors. Neuroimaging research has revealed atypical connectivity in brain regions associated with emotion regulation and sensory processing, notably the anterior insular cortex (AIC) and the prefrontal cortex [19,20]. For instance, individuals with misophonia exhibit exaggerated responses to trigger sounds in the AIC, a key region in processing emotions and interoceptive signals [19]. Additionally, heightened connectivity between the AIC and auditory cortices has been observed, further supporting the role of the AIC in processing emotional and sensory input during exposure to misophonic triggers [21,22]. These findings suggest that misophonia shares neurobiological characteristics with other conditions, such as anxiety disorders and obsessive-compulsive personality disorder, indicating that broader emotional and regulatory processes may contribute to its manifestation and maintenance [19,20].

Emotion regulation, defined as the strategies and processes involved in monitoring, evaluating, and modifying emotional reactions [23], may play an important role in the experience and management of misophonia. Characterized by extreme autonomic and emotional responses to typically innocuous auditory stimuli [2,21,24,25], misophonia may be influenced by difficulties in emotion regulation. Research indicates that deficits in emotion regulation are associated with the development and exacerbation of misophonia symptoms, as the inability to manage intense emotional responses to misophonic triggers appear to increase both the severity and impact of the condition [24,26–28]. Moreover, strategies like avoidance and escape behaviors, though negatively reinforced as relieving in the short-term, may unintentionally contribute to worsening impairment in functioning over time, amplifying distress and anxiety [24,28,29].

The Difficulties in Emotion Regulation Scale (DERS) is a widely used measure assessing emotion regulation across six dimensions: Nonacceptance of Emotional Responses, Difficulties Engaging in Goal-Directed Behavior, Impulse Control Difficulties, Lack of Emotional Awareness, Limited Access to Emotion Regulation Strategies, and Lack of Emotional Clarity [30]. This measure has been applied across various clinical populations to examine how deficits in emotion regulation contribute to psychopathology [30], including disorders characterized by intense emotional responses and challenges in managing them, such as borderline personality disorder [31], generalized anxiety disorder [32], substance use disorders [33,34], social anxiety [35], post-traumatic stress disorder [36], and bipolar disorder [37,38]. Given the pronounced difficulties with emotion regulation observed in misophonia, the DERS has begun to be used in studies on this condition to assess how specific regulatory deficits may exacerbate misophonic reactions to trigger sounds [26,28]. These applications underscore the potential of the DERS in elucidating the role of emotion regulation in misophonia, particularly how deficits in specific facets of emotion regulation, such as acceptance and impulse control, may fuel maladaptive responses to auditory triggers.

Anger is a central emotional response associated with misophonia. Individuals with misophonia often report that trigger sounds can lead to immediate and overwhelming feelings of anger, ranging from mild irritation to explosive rage [1,6,8,9,39–42]. Anger is frequently accompanied by physiological arousal, such as increased heart rate, muscle tension, and changes in blood pressure, which can amplify the emotional experience [43,44]. The intensity of these reactions may reflect challenges in emotional regulation when faced with specific auditory triggers. Although physical violence is rare among those with misophonia, the anger elicited by trigger sounds often leads to a range of behavioral responses. These responses can include indirect expressions of aggression, such as verbal outbursts or shutting down communication, as well as non-physical confrontations [42,45]. Research suggests that individuals with misophonia frequently experience thoughts of aggression or imagine aggressive responses, but are highly unlikely to engage in violence when triggered [46]. For example, some may respond with verbal aggression or by avoiding social situations, communicating indirectly, or making demands of others, which can lead to misunderstandings, conflicts, and social isolation [1,9,39,40]. This potential cycle of anger and social repercussions underscores the importance of better characterizing the relationship between anger and emotion dysregulation in misophonia to inform intervention development that effectively targets these challenges.

Anxiety is another core emotional response commonly reported by individuals with misophonia, often linked to the anticipation or presence of trigger sounds. Unlike anger, which tends to provoke immediate and intense reactions, anxiety related to misophonia may build gradually, leading to chronic stress and pervasive worry about encountering trigger sounds in various environments [40,47]. This anxiety may be characterized by heightened vigilance and a fear of potential exposure to these sounds, which can significantly disrupt daily functioning [5,48,49]. Understanding anxiety in the context of misophonia is important, as it can influence how individuals react to trigger sounds and affect their social and emotional well-being. For some, heightened baseline anxiety may make them more sensitive to emotional and sensory stimuli, which may increase the likelihood of developing misophonia [26,40,49]. Conversely, the chronic stress and social difficulties associated with misophonia can exacerbate existing anxiety, leading to a cycle of anticipatory worry and avoidance [5–7,40,50–54]. This relationship between anxiety and misophonia highlights the need for therapeutic approaches that address both anxiety directly and emotional regulation broadly to help individuals better cope with distressing symptoms.

To gain a more comprehensive understanding of how emotion tends to be regulated in people with misophonia, it is important to examine how misophonia severity is related to various facets of emotion regulation and common, distressing, and impairing affects (anger and anxiety) experienced in the condition. Network analysis provides a powerful tool for visualizing these relationships, helping to identify which affects and emotion regulation difficulties are most closely associated with misophonia severity. Previous research has demonstrated the value of symptom network models in capturing the relationships among sensory sensitivities and clinical traits, particularly highlighting how misophonia severity is linked to disruptions in sensory processing and attentional mechanisms, alongside various comorbidities [55]. Building on this work, our study extends these findings by focusing on how specific facets of emotion regulation interact with misophonia severity. This novel extension of the literature allows us to map more nuanced interrelations between emotion regulation processes and misophonia severity, potentially revealing key regulatory targets for intervention.

This approach aligns with the principles of process-based therapy (PBT) [56], which aims to understand the dynamic relationships among biopsychosocial processes that contribute to individual distress. PBT emphasizes flexibility in targeting specific processes relevant to each individual, rather than applying fixed diagnostic categories. By using network analysis, we can map the interrelations among emotion regulation difficulties, anger, and anxiety, providing a clearer picture of the key processes that most account for misophonia severity. This can help facilitate identification of critical intervention targets, particularly in areas where emotion dysregulation and heightened affects, like anger and anxiety, may amplify distress. In doing so, network analysis helps pinpoint specific areas where tailored interventions might reduce symptom intensity and improve emotion management [57]. This study uses network analysis to explore these relationships and identify potential treatment targets to address both regulatory and emotional challenges in misophonia.

## Materials and methods

### Participants

This study included a sample of 205 adults (average age = 37.8 years; females = 72.7%; see Table 1 for demographic information) residing in the United States with self-reported misophonia symptoms. Participants were recruited via a REDCap link posted on the Duke Center for Misophonia and Emotion Regulation website [58]. The Duke Health Institutional Review Board (IRB) approved study procedures and all participants provided written informed consent before participation. Study visits were conducted remotely between December 9, 2019 and July 23, 2024, followed by several self-report questionnaires that participants were asked to fill out on their own (see below). Eligibility criteria excluded individuals with a current psychotic disorder, mania, or anorexia nervosa. These criteria were confirmed through a phone screening using the Mini International Neuropsychiatric Interview (M.I.N.I.) after completion of the online screening form in REDCap.

**Table 1:** Demographic characteristics of the current sample.

Characteristic	*n*	%
***Age in years (M, SD)***	37.8	12.7
**Sex**		
Male	56	27.3
Female	149	72.7
**Gender Identity**		
Male	56	27.3
Female	144	70.2
Genderqueer	1	0.5
Other	2	1.0
Did not disclose	2	1.0
**Sexuality**		
Straight	160	78.0
Gay	12	5.9
Bisexual	16	7.8
Something else	10	4.9
Don’t know	7	3.4
**Race**		
White	161	78.5
Native American	2	1.0
African American	11	5.4
Chinese or Chinese American	6	2.9
Other Asian	7	3.4
Other	6	2.9
More than one race	12	5.9
**Hispanic/Latinx**		
No	174	84.9
Yes	31	15.1
**Income Level**		
0-$10,000	23	11.2
$10,001 - $20,000	8	3.9
$20,001 - $40,000	21	10.2
$40,001 - $65,000	38	18.5
$65,000 - $100,000	34	16.6
More than $100,000	81	39.5
**Marital Status**		
Single	76	37.1
Widowed	1	0.5
Married	90	43.9
Separated	4	2.0
Divorced	10	4.9
Living with Partner	22	10.7
Missing	2	1.0
N = 205		

### Measures

#### Misophonia Questionnaire (MQ).

The Misophonia Questionnaire (MQ) [7] is a 17-item, widely used [50,59–61], self-report measure that assesses the severity and impact of misophonia symptoms across multiple dimensions. Participants completed the MQ as part of the online screen posted on our Center’s website before enrolling in the study visit. The MQ measures impairment with a single item ranging from 0 to 15, with scores of 6 or higher considered clinically significant. In the current study, 88% of participants had an MQ impairment score of 6 or higher, indicating that the majority of the sample consists of individuals with clinically significant misophonia symptoms. This focus aligns with the study’s aim of exploring misophonia among those experiencing substantial functional impairment. However, we also included participants with scores below this threshold to capture a broader range of misophonia presentations, including those who may have significant symptom severity on other facets of misophonia as captured in the Duke Misophonia Questionnaire (DMQ), but who do not meet the threshold for clinically significant impairment.

#### Duke Misophonia Questionnaire (DMQ).

Duke Misophonia Questionnaire (DMQ) [62] is an 86-item psychometrically validated self-report tool developed in an English-speaking sample using factor analytic procedures and item response theory (IRT), which models item performance across the underlying trait continuum to optimize scale precision and detect local item misfit [63,64]. This study used four subscales of the DMQ to capture the different facets of misophonia: Affective Responses (8 items), Physiological Responses (5 items), Cognitive Responses (10 items), and Impairment (12 items). The Affective Responses subscale measures emotional reactions to trigger sounds, such as disgust, hostility, and frustration. The Physiological Responses subscale assesses physical reactions, including trembling and heart pounding. The Cognitive Responses subscale examines cognitive reactions to triggers (e.g., “I cannot handle this”, “Everything is awful,” and “How do I make this sound stop?”), while the Impairment subscale evaluates the impact of misophonia on daily functioning. Impairment scores on the DMQ range from 0 to 48, with clinical classifications as follows: 0–13 (minimal to mild impairment), 14–38 (moderate impairment), and 39–48 (severe to very severe impairment). In the current sample, the mean clinical impairment score was 8.89 (SD = 6.40), suggesting that impairment levels were mild. The Symptom Severity composite score, combining the Affective, Physiological, and Cognitive subscales, ranges from 0 to 83. Scores greater than 41 are indicative of clinically significant misophonia symptoms. In our sample, the mean symptom severity composite score was 34.00 (SD = 18.44).

Despite the MQ’s ability to identify clinically significant impairment with one item, the DMQ was selected for use in the network analysis due to its comprehensive structure and its capacity to yield inferences about symptom severity and functional impairment. The DMQ includes a composite Symptom Severity score along with an Impairment subscale, enabling assessments of both the broader symptomatology and the functional impact of misophonia, which are particularly relevant for this study. Although the DMQ contains multiple subscales, we specifically utilized the Affective Responses, Physiological Responses, Cognitive Responses, and Impairment subscales, as these domains are the most pertinent for capturing misophonia symptom severity and its impact on daily functioning. This study examines the DMQ from an aggregated perspective rather than analyzing each subscale individually. However, the selection of these key subscales ensures that the DMQ offers a detailed representation of the condition’s impact across essential areas of functioning. Furthermore, the DMQ has been psychometrically validated using both factor analytic procedures and item response theory (IRT), providing robust evidence for its reliability and validity in measuring misophonia symptoms across diverse populations [62]. The scale demonstrated good internal consistency in this sample, with a Cronbach’s alpha of α = 0.76 and McDonald’s omega of 0.88.

#### Difficulties in Emotion Regulation Scale (DERS).

The Difficulties in Emotion Regulation Scale (DERS) [30] is a 36-item self-report measure assessing six trait level domains of emotion regulation difficulties: Nonacceptance of Emotional Responses (Nonaccept), Difficulty Engaging in Goal-Directed Behavior (Goals), Impulse Control Difficulties (Impulse), Lack of Emotional Awareness (Awareness), Limited Access to Emotion Regulation Strategies (Strategies), and Lack of Emotional Clarity (Clarity). Items are rated on a 5-point Likert scale, from 1 (almost never) to 5 (almost always), with higher scores indicating greater difficulties in emotion regulation. The mean total DERS score in the current sample was 79.29 (SD = 20.50). Subscale means were as follows: Nonacceptance, 13.28 (SD = 5.60); Goals, 14.74 (SD = 4.43); Impulse, 9.97 (SD = 3.80); Awareness, 14.16 (SD = 4.43); Strategies, 16.55 (SD = 6.51); and Clarity, 10.59 (SD = 3.55). The scale demonstrated strong internal consistency in this sample, with a Cronbach’s alpha of α = 0.80 and McDonald’s omega of 0.90. Subscale-level internal consistency was also high: Nonacceptance (α = 0.919; ω = 0.938), Goals (α = 0.897; ω = 0.917), Impulse (α = 0.843; ω = 0.895), Awareness (α = 0.804; ω = 0.866), Strategies (α = 0.896; ω = 0.920), and Clarity (α = 0.837; ω = 0.874).

#### Generalized Anxiety Disorder (GAD-7).

The Generalized Anxiety Disorder-7 (GAD-7) [65] is a 7-item self-report measure assessing the severity of generalized anxiety disorder symptoms. Each item is rated on a 4-point Likert scale from 0 (not at all) to 3 (nearly every day), with total scores ranging from 0 to 21. Higher scores indicate greater severity of anxiety symptoms. The GAD-7 has established clinical cutoffs indicating minimal (0–4), mild (5–9), moderate (10–14), and severe (15–21) anxiety symptoms [65]. In this sample, the mean GAD-7 score was 6.68 (SD = 5.17), corresponding on average to mild anxiety symptoms. The scale demonstrated strong internal consistency in this sample, with a Cronbach’s alpha of α = 0.89 and McDonald’s omega of 0.91.

#### Clinically Useful Anger Outcome Scale (CUANGOS).

The Clinically Useful Anger Outcome Scale (CUANGOS) [66] is a 5-item self-report that measures the frequency and intensity of anger episodes and their impact on functioning over the past week. Items are rated on a 5-point Likert scale, from 0 (not at all) to 4 (extremely), with higher scores reflecting greater anger and its detrimental effects on daily life. The CUANGOS lacks formally established clinical cutoffs; however, based on prior analyses and clinical experience, scores of 0–2 reflect no/minimal anger, 3–5 indicate mild anger, 6–9 indicate moderate anger, and scores of 10 or above reflect severe anger symptoms [66]. Only the composite score was used in this study, with a mean score of 3.65 (SD = 4.13), indicating mild anger severity on average. The scale demonstrated strong internal consistency in this sample, with a Cronbach’s alpha of α = 0.87 and McDonald’s omega of 0.91.

### Study procedures

All study procedures were approved by the Duke Health Institutional Review Board (IRB), and participants provided written informed consent prior to participation. Study visits involved administration of the Structured Clinical Interview for DSM-5 [67] by trained assessors, followed by several self-report questionnaires completed independently by participants. Participants received $75 USD in compensation upon completion of the clinical interviews and self-report assessments.

### Statistical analysis

All analyses were conducted using R Studio (Version 2023.06.1 + 524). To address the 4.6% of data that was missing, multiple imputation was performed using the *mice* package, resulting in complete datasets for all 205 participants. Predictive mean matching (PMM) was used as the imputation method due to its robustness in preserving data distributions and suitability for handling numeric variables with skewed distributions. We conducted five imputations, each with five iterations (the default in the *mice* package), to ensure convergence and stability of imputation estimates.

Misophonia impairment was assessed using both the Duke Misophonia Questionnaire (DMQ) and the Misophonia Questionnaire (MQ). On average, impairment measured by the DMQ was mild, whereas most participants (88%) reported clinically significant impairment on the MQ. To facilitate direct comparison between the two measures, we normalized both scores to a 0–100 percentage scale, accounting for their differing maximum values (DMQ: 48-point maximum; MQ: 15-point maximum). The resulting delta, representing the percentage difference between DMQ and MQ impairment scores, allowed us to compare impairment levels on the same unit scale.

To investigate the relationships among misophonia severity, emotion regulation, anger, and anxiety, the first step in the network analysis involved deriving a composite score for misophonia severity. Given the modest sample size and the statistical requirements of network analysis (i.e., maintaining model stability and parsimony), we used a composite misophonia severity score by summing standardized z-scores of DMQ symptom and impairment subscales [68]. We recognize, however, that future studies with larger samples may benefit from modeling individual DMQ subscales separately to capture potentially distinct misophonia dimensions and their differential associations with emotion regulation. This composite measure was included as a node in the network, along with nodes representing emotion regulation subscales (DERS: Nonacceptance, Goals, Impulse, Awareness, Strategies, Clarity), anger (CUANGOS), and anxiety (GAD-7), resulting in nine nodes included in the network. The network structure was estimated using a Gaussian Graphical Model (GGM) via the *bootnet* package [69]. This model examines the partial correlations among variables, where each node represents a variable, and the edges reflect the partial correlation coefficients between pairs of nodes. We used the graphical least absolute shrinkage and selection operator (gLASSO) for regularization, a technique that reduces the likelihood of false positives by shrinking small partial correlations towards zero. The extended Bayesian information criterion (EBIC) was used to set the tuning parameter (gamma value set at 0.5) for this regularization, selected to balance sensitivity and specificity, reducing the risk of false-positive edges while retaining relevant connections within the network [70]. We also identified communities within the network using the Walktrap algorithm from the *igraph* package to better understand the underlying structure of the relationships among emotion regulation, misophonia severity, anger, and anxiety. This algorithm uses random walks to detect communities within the network, grouping nodes that are more likely to be connected into a community. The Walktrap community detection algorithm was chosen because of its robustness in identifying meaningful node groupings through random walk methods, optimizing the interpretability of the network’s underlying structure.

Centrality indices, strength centrality and one- and two-step expected influence, were calculated to determine the importance of each node in the overall network. Strength centrality measures the extent to which a node is directly connected to other nodes, calculated by summing the absolute weights of all edges connected to that node. Expected influence (EI) considers both direct and indirect connections of a node up to two steps away. One-step EI calculates the sum of all direct edge weights (positive and negative, rather than the absolute values) connected to a node, while two-step EI extends this calculation to include the node’s influence on its immediate neighbors (i.e., nodes) and those neighbors’ connections, allowing for a more detailed understanding of a node’s overall impact within the network [71,72]. Using both strength centrality and expected influence helps identify critical nodes that may be pivotal for targeted interventions in misophonia, accounting for their immediate and extended influence within the network. Centrality metrics (strength and expected influence) were selected due to their demonstrated efficacy in identifying clinically relevant nodes within psychological networks. Bridge centrality was used to evaluate the role of nodes in connecting different communities within the network [73]. Bridge strength centrality quantifies how strongly a node is connected to nodes in other communities by summing the absolute weights of these inter-community edges. In contrast, bridge expected influence (EI) measures the extent to which a node can impact nodes in other communities, considering both direct and indirect connections. Together, these metrics help identify key nodes that may serve as effective intervention targets, potentially influencing broader network processes in misophonia. To assess the robustness and stability of centrality indices and network structure, we conducted non-parametric bootstrapping (case-dropping) with 2,500 resamples using the *bootnet* package with Spearman correlations [69]. Bootstrapping was executed in parallel on eight computing cores (nCores = 8) to enhance computational efficiency.

## Results

**Fig 1:**
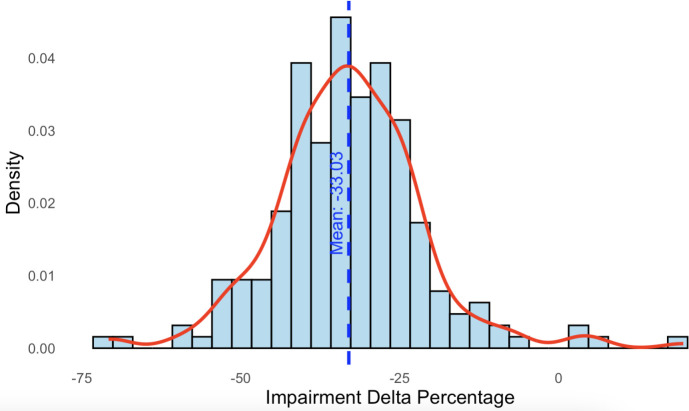
Distribution of misophonia impairment delta percentage between DMQ and MQ measures. This density plot shows the distribution of misophonia impairment delta percentages (percentage impairment DMQ minus percentage impairment MQ) for each participant. The plot highlights the variability in reported impairment between the two measures. On average, participants reported lower impairment scores on the DMQ compared to the MQ, with a mean delta of −33%, as indicated by the blue dashed line.

The distribution of misophonia impairment delta percentages (percentage impairment DMQ minus percentage impairment MQ) for each participant is depicted in the density plot shown in Fig 1. The delta scores, representing the difference between normalized impairment scores on the DMQ and the MQ, ranged from −70% to 20%, with a mean delta of −33%. This density plot highlights the variability in impairment scores between the two measures, showing how on average, participants reported lower impairment scores on the DMQ compared to the MQ.

Normality was assessed, with skewness and kurtosis values all less than 2, in line with established guidelines [74]. Table 2 summarizes the means, standard deviations, skewness, and kurtosis values for all nodes included in the network analysis. Although no formal power analysis exist for network analysis, prior work has suggested that the sample size, or observations in a network, should exceed the number of potential parameters [69]. Our sample size of 205 exceeded the number of potential estimated parameters of 36.

**Table 2:** Descriptive statistics for network nodes. This table presents the means, standard deviations, skewness, and kurtosis values for each of the nodes included in the network analysis. These values show the distribution characteristics of the variables used in the network model. Skewness and kurtosis values are all below 2, indicating acceptable levels of normality in the data, consistent with established guidelines.

Variable	Mean	SD	Skewness	Kurtosis
Anxiety	6.68	5.17	0.79	−0.12
Nonaccept	13.28	5.60	1.05	0.59
Goals	14.74	4.43	0.26	−0.61
Impulse	9.97	3.80	1.41	1.87
Aware	14.16	4.43	0.32	−0.50
Strat	16.55	6.51	0.87	0.25
Clarity	10.59	3.55	0.81	0.30
Anger	3.65	4.13	1.37	1.62
DMQ	0.00	1.81	0.21	−0.46

**Fig 2:**
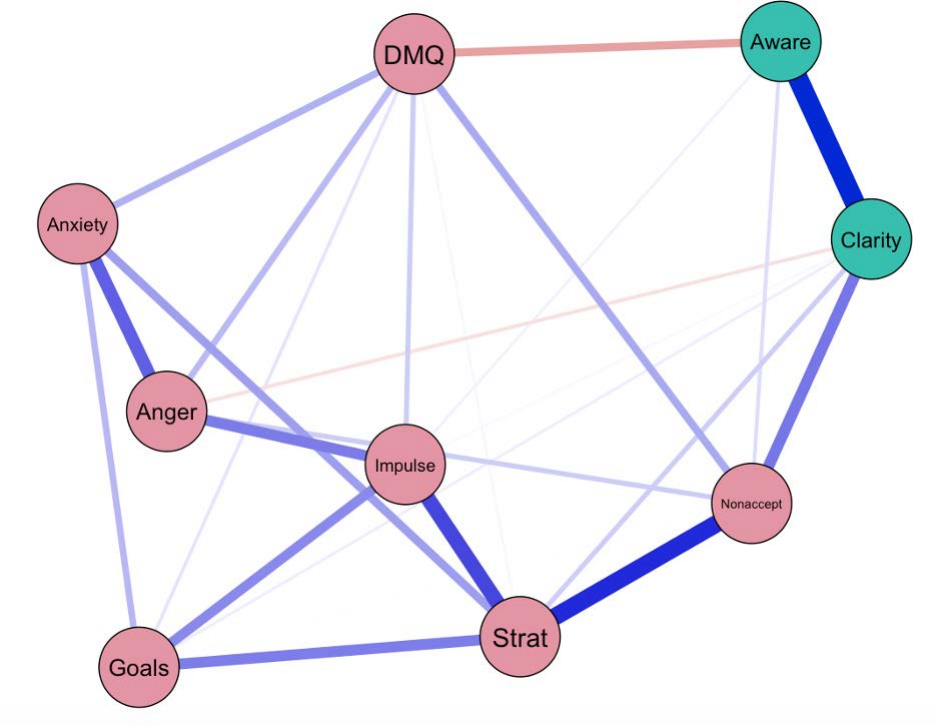
Visual representation of the network. The figure visualizes the relationships among the variables in the network. Nodes represent variables, and edges represent partial correlations between the nodes. Blue edges indicate positive associations, and red edges indicate negative associations. Thicker edges correspond to stronger associations. Detected communities are color-coded, reflecting the underlying structure of the relationships between anger, anxiety, emotion regulation, and misophonia severity. Pink nodes represent nodes that are part of the “Emotion Dysregulation and Misophonia” community and green nodes represent nodes that are part of the “Emotional Clarity and Awareness” community.

Our resulting network included 23 (64%) significant associations, evidencing a network with a dense level of connectivity. In Fig 2, blue edges indicating positive associations and red edges indicating negative associations. The thickness of each edge corresponds to the strength of the association between nodes, with thicker lines signifying stronger associations. The detected communities, derived from the measures used in this analysis, are color-coded and depicted within the network in Fig 2. Our analysis using the Walktrap algorithm identified two distinct communities within the network, reflecting underlying patterns based on the subscales of the selected measures. We name these communities “Emotion Dysregulation and Misophonia” (EDM) and “Emotional Clarity and Awareness (ECA).” The EDM community included nodes representing “Anxiety,” “Nonacceptance,” “Anger,” “Impulsivity,” “Goals,” “Strategies,” and “Misophonia.” The ECA community comprised nodes corresponding to “Clarity” and “Awareness.” As shown in S1 and S2 Figs, “Clarity” and “Nonacceptance” demonstrated the highest bridge strength centrality and bridge one- and two-step expected influence within the network, indicating their role in linking the EDM and ECA communities.

**Fig 3:**
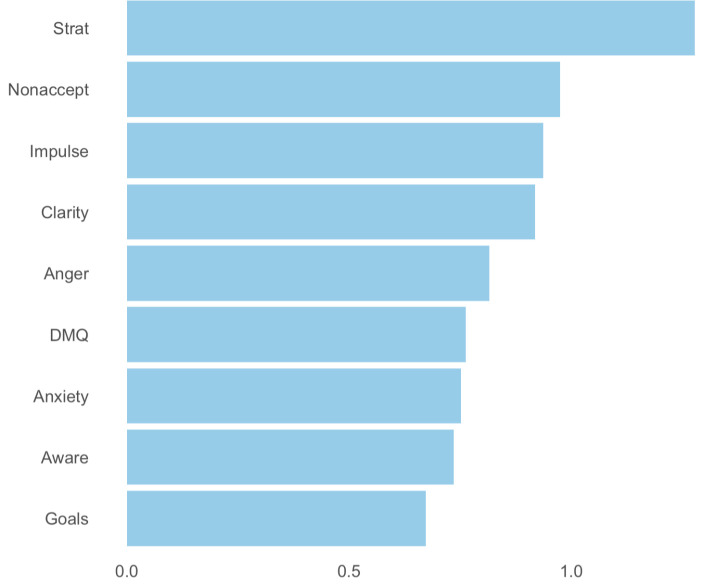
Strength centrality of network nodes. This bar chart depicts the strength centrality values for each node in the network, ordered from highest to lowest strength. Strength centrality represents the sum of the absolute weights of edges connected to each node, indicating the direct influence of the node on others in the network. “Strategies,” “Nonacceptance,” and “Impulsivity” demonstrate the highest strength, suggesting these variables are central to the network structure.

**Fig 4:**
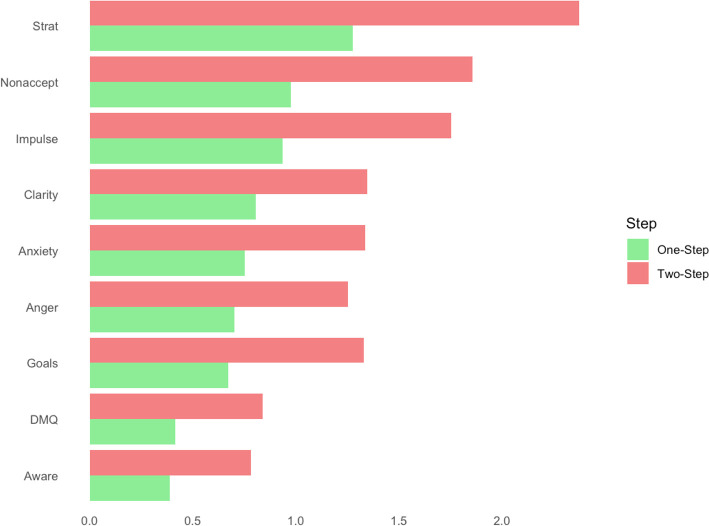
One-step and two-step expected influence of network nodes. The figure presents the one-step and two-step expected influence values for each node, with nodes sorted by one-step expected influence. One-step expected influence reflects the sum of direct connections (both positive and negative), while two-step expected influence accounts for indirect connections via adjacent nodes. “Strategies,” “Nonacceptance,” and “Impulsivity” demonstrate the highest expected influence, suggesting that these variables have both direct and extended impacts on other nodes in the network.

As shown in Fig 3, strength centrality was calculated for each node in the network, with the nodes representing “Strategies,” “Nonacceptance,” and “Impulsivity” demonstrating the highest strength centralities, with values of 1.28, 0.97, and 0.94, respectively. The strength centrality of “Strategies” was significantly greater than the strength centrality of the 7 (87.5%) other nodes in the network; however, it was not significantly greater than “Nonacceptance.” (S3 Fig in S1 File). Fig 4 depicts both one-step and two-step EI for each node. “Strategies,” “Nonacceptance,” and “Impulsivity” also demonstrated the highest one- and two-step EI with values of 1.28/ 2.37, 0.97/ 1.86, and 0.94/ 1.75 respectively. The EI of “Strategies” was also significantly greater than all other nodes’ EI centralities besides “Nonacceptance”.

The correlation stability coefficients (CS-coefficients) for node strength and EI were calculated at 0.52 and 0.60, respectively, surpassing the recommended threshold of 0.50, which supports the reliability of interpreting these nodes’ importance [69]. Further, the bootstrapped edge-weight accuracy test revealed that the strongest edges were all among nodes of the same measure, including between “Awareness” and “Clarity,” “Nonacceptance” and “Strategies,” and “Impulsivity” and “Strategies,” with edge-weights of 0.47, 0.41, and 0.34, respectively. Additionally, when evaluating the edges between different measures, the edges between “Anger” and “Anxiety,” “Impulsivity” and “Anger,” and “Anxiety” and “Strategies” were the strongest, with edge-weights of 0.30, 0.24, and 0.18, respectively. Within the context of the DMQ, the strongest associations were observed with nodes representing “Awareness”, “Nonacceptance,” “Anxiety,” and “Anger” with edge-weights of −0.17, 0.16, 0.15, and 0.13, respectively. Table 3 provides a full list of non-zero edge weights.

**Table 3:** Non-zero edge weights in the network. This table lists the unique non-zero edge weights detected in the network, representing partial correlations between pairs of nodes. Stronger connections between nodes are denoted by higher absolute edge weights. These associations provide insights into how closely different aspects of emotion regulation, anger, anxiety, and misophonia severity are linked within the network.

Node 1	Node 2	Weight
Clarity	Aware	0.4739
Strat	Nonaccept	0.4081
Strat	Impulse	0.3446
Anger	Anxiety	0.2950
Clarity	Nonaccept	0.2522
Anger	Impulse	0.2430
Strat	Goals	0.2400
Impulse	Goals	0.2168
Strat	Anxiety	0.1791
DMQ	Aware	−0.1737
DMQ	Nonaccept	0.1577
DMQ	Anxiety	0.1457
Goals	Anxiety	0.1312
DMQ	Anger	0.1289
Anger	Nonaccept	0.0927
DMQ	Impulse	0.0927
Clarity	Strat	0.0912
Aware	Nonaccept	0.0612
Anger	Clarity	−0.0558
DMQ	Goals	0.0491
Clarity	Goals	0.0324
Aware	Impulse	0.0267
DMQ	Strat	0.0148
Clarity	Impulse	0.0124
Goals	Nonaccept	0.0026

## Discussion

This study used network analysis to explore the relationships among misophonia severity, emotion regulation, anxiety, and anger. Although limited access to emotion regulation strategies was the most influential node across the network, its direct link with misophonia severity was minimal. Nonacceptance of emotions, anxiety, and anger showed modest yet the strongest positive associations with misophonia severity, while goal-directed behavior, strategies, impulsivity, and emotional clarity did not show significant direct associations. Notably, lower emotional awareness was linked to reduced misophonia severity. The network analysis revealed two distinct communities: “Emotional Clarity and Awareness (ECA),” including awareness and clarity, and “Emotion Dysregulation and Misophonia (EDM),” comprising anxiety, anger, misophonia severity, and other emotion regulation difficulties. Clarity and nonacceptance had the highest bridge strength and expected influence (EI), serving as key connectors between the two communities.

### Nonacceptance and misophonia severity

Our results demonstrated that nonacceptance of emotions, characterized by difficulty accepting emotional states without judgment, may play a more significant role in misophonia severity than previously suggested. A previous preliminary study found a relatively weak bivariate relationship between nonacceptance and misophonia severity [26], but we observed that nonacceptance had the largest direct partial correlation with misophonia severity as well as the second highest strength centrality. This suggests that nonacceptance may not only influence misophonia severity but also interact with other critical processes in emotion dysregulation. Furthermore, nonacceptance of emotions showed the highest bridge strength and expected influence (EI) within the EDM community, emphasizing its role in connecting emotional dysregulation and misophonia severity to emotional awareness and clarity. Additionally, our findings showed a positive association between nonacceptance and heightened anger, suggesting that nonacceptance may intensify emotional reactivity, particularly anger toward misophonic triggers. This interaction could lead to a reinforcing cycle of distress, where nonacceptance amplifies immediate emotional responses and hinders cognitive processing of these emotions, in line with prior research indicating that nonacceptance can intensify distress and contribute to cycles of maladaptive emotional reactivity [27,75,76]. Addressing nonacceptance in therapeutic interventions could help break this cycle by promoting nonjudgmental acceptance, potentially reducing the emotional burden associated with misophonia [77–83].

### Anger and misophonia severity

Anger showed a modest yet significant positive association with misophonia severity, aligning with previous research suggesting anger is a prominent emotional state associated with misophonia and contributes to the distress experienced [1,9,39–42]. Although the CUANGOS measures general anger frequency and intensity [66] rather than anger directly tied to misophonic triggers, its alignment with intense irritation and aggressive impulses reported in misophonia suggests that individuals with higher misophonia severity may also report heightened anger. This association, however, should be interpreted with caution, as it remains unclear whether CUANGOS scores capture anger specifically in response to misophonic triggers or reflect a broader tendency toward anger in the context of misophonia. The anger response may arise partly because misophonic triggers interfere with the individual’s ability to achieve context-dependent goals—anger often surfaces when task completion is obstructed or threatened. For instance, when a trigger sound disrupts a person’s focus or impedes their intended actions, it may elicit an anger response that evolutionarily signals the need to address the interference. Regardless of whether this anger reflects a general tendency or is context-specific, addressing anger in therapeutic interventions could help reduce emotional dysregulation, improve coping responses to triggers, and ultimately lessen overall distress [57,84,85].

### Anxiety and misophonia severity

Anxiety showed a modest but significant positive association with misophonia severity, aligning with prior research [5–7,40,50,51,53,54]. The GAD-7 captures symptoms like excessive worry and restlessness, which may relate to the heightened vigilance often observed in misophonia [65]. In this context, anxiety may manifest as hypervigilance, a state of arousal that increases sensitivity to potential misophonic triggers. This heightened arousal could impair the ability to regulate emotions effectively, intensifying distress and interfering with cognitive processes such as sustained attention, memory consolidation, and learning. For individuals with misophonia, the anticipation of encountering triggers may further amplify distress, increasing vigilance and straining attentional resources. This heightened state can disrupt the application of adaptive emotion regulation strategies [29], making it more difficult to manage reactions during exposure to triggers. These disruptions often lead to difficulty focusing and processing information effectively, contributing to functional impairments in environments that demand sustained attention and goal-directed behavior, such as work or school [1,86]. Furthermore, this interference could intensify anger responses, complicating the regulation process and contributing to a cycle of misophonic distress. Addressing anxiety and hypervigilance, along with their downstream effects on attentional and regulatory processes, may help mitigate the functional impairments and distress associated with misophonia.

### Emotional awareness and misophonia severity

The negative association between lack of emotional awareness and misophonia severity challenges the conventional emphasis on emotional awareness for distress management [87]. Our findings suggest that lower awareness of emotional states might reduce reactivity to misophonic triggers. Prior studies link fixation on triggers and deficits in nonjudgmental awareness to increased misophonia severity [3,21,81,83]. Heightened emotional awareness could worsen misophonia by intensifying focus on distressing triggers, while reduced awareness might mitigate this hyperfocus, lessening distress. Interestingly, our network analysis revealed that nonacceptance has the highest bridge strength and expected influence within the EDM community, representing the node most closely associated with the ECA community. Our network analysis also revealed nonacceptance as the node most closely associated with the ECA community, suggesting an interaction among emotional awareness, clarity, and acceptance. This implies that emotional awareness alone may not suffice for managing reactivity in misophonia; instead, combining awareness with nonjudgmental acceptance could be crucial in reducing hyperfocus on emotional experiences [26,83,87]. Neurophysiological research supports this interpretation, showing that misophonia is often accompanied by maladaptive attentional processes, such as heightened sensitivity to auditory stimuli, which complicates emotional regulation [20,88–91]. Thus, in misophonia, increased emotional awareness may enhance maladaptive focus on triggers, amplifying distress.

### Community analysis: Separate “Emotional Clarity and Awareness” (ECA) community

The ECA community, including the awareness and clarity subscales, emerged separately from all the other nodes in the network. This distinction aligns with existing literature, where awareness often does not cluster with other DERS subscales as it focuses on attention to emotions rather than regulation [92–94]. Clarity, which involves understanding and differentiating emotional states, is closely related to awareness [94]. Studies suggest that while awareness refers to noticing emotions, clarity involves making sense of them [95,96]. This relationship likely explains their clustering together, distinct from the other nodes in the network. In the context of misophonia, the separation of ECA from other emotion regulation difficulties suggests that awareness and clarity may play a different role. They contribute to recognizing and understanding emotional responses rather than regulating them. This aligns with the notion that individuals with misophonia might be acutely aware of their emotional reactions to trigger sounds but struggle with managing these intense emotions.

### Implications for clinical practice

The network analysis in this study highlights the interconnected nature of emotional regulation difficulties, anger, and anxiety in misophonia, emphasizing the utility of a process-based therapeutic approach [56]. Consistent with PBT principles, this approach identifies dynamic processes that sustain distress, providing a flexible framework for targeted interventions. The use of a network model in this context adds precision by mapping the interrelations among emotional processes, allowing for the identification of specific nodes—nonacceptance, anger, and anxiety—that showed the strongest direct partial correlations with misophonia severity. These nodes emerge as critical points for intervention. To facilitate clinical translation of these findings, Table 4 summarizes the primary network nodes, their clinical implications, and corresponding evidence-based intervention approaches.

**Table 4:** Intervention targets and recommended treatments for misophonia. This table summarizes the core nodes identified in the network analysis, their clinical relevance, and corresponding evidence-based interventions, including ACT, CBT, DBT, Mindfulness-Based Interventions, and Unified Protocol modules, to guide individualized treatment for misophonia.

Node	Clinical Implication	Recommended Therapeutic Approaches
Nonacceptance of Emotions	Difficulty accepting emotional states intensifies distress, particularly anger responses, creating a cycle of reactivity.	Acceptance and Commitment Therapy (ACT); Mindfulness-Based Interventions; Unified Protocol (UP), including Mindful Emotional Awareness and Countering Emotional Behaviors Modules.
Anger	Intense reactions to misophonic triggers contribute to emotion dysregulation and impulsive behaviors.	Dialectical Behavior Therapy (DBT) (STOP and Opposite Action skills); Cognitive Restructuring; Mindfulness-Based Interventions; Unified Protocol (UP) (Cognitive Flexibility, Countering Emotional Behaviors, and Emotion Exposure)
Anxiety	Heightened vigilance and anticipatory distress exacerbate emotional sensitivity, attentional disruption, and avoidance behaviors, amplifying misophonia severity.	Cognitive-Behavioral Therapy (CBT): Cognitive Restructuring, Attentional Shifting, and Distress Tolerance; Unified Protocol (UP) (Cognitive Flexibility, Facing Physical Sensations, and Countering Emotional Behaviors to address both anticipatory avoidance and hyperarousal)
Emotional Awareness	Reduced emotional awareness might mitigate distress through decreased focus on emotional reactions, but optimal management may require combining awareness with acceptance strategies to avoid maladaptive hyperfocus	Unified Protocol (UP)—Mindful Emotional Awareness combined with acceptance-oriented exercises throughout treatment; Mindfulness-Based Cognitive Therapy.
Emotional Clarity	Enhanced clarity aids in understanding emotions but does not directly regulate them; plays a role in recognizing triggers and emotional responses.	Psychoeducation on emotion identification and differentiation; Unified Protocol (UP) (Understanding Your Emotions, Mindful Emotional Awareness, and Cognitive Flexibility to promote clearer emotional understanding)

One can consider integrating interventions like fostering emotional acceptance, addressing anger, and reducing anxiety and avoidance within an evidence-based, process-based treatment framework. Such strategies offer promising candidates for reducing symptom severity and improving functioning in individuals with misophonia.

#### Enhancing emotional acceptance.

Addressing nonacceptance of emotions is key in misophonia treatment, as nonacceptance is linked to increased distress and maladaptive coping strategies like avoidance and aggression [24,27,75,76,79]. Therapeutic approaches such as Acceptance and Commitment Therapy (ACT) and mindfulness-based interventions can foster nonjudgmental acceptance, guiding patients to respond to emotional triggers with less reactivity [79–83]. By cultivating acceptance, patients may view emotions as temporary, reducing the intensity and frequency of reactions to triggers.

While increased emotional awareness alone may heighten focus on distressing triggers, integrating acceptance may help mitigate this effect. The Unified Protocol (UP) for misophonia [97] includes modules like “Mindful Emotional Awareness” that combine awareness with an accepting attitude toward emotions. This approach encourages patients to observe their emotions in the present moment without judgment or impulsive reactions, reducing hypervigilance and fostering emotional acceptance. The “Countering Emotional Behaviors” module complements this by helping patients identify and modify avoidance behaviors stemming from nonacceptance of emotions, encouraging actions that align with personal values. By directly targeting nonacceptance, these strategies can improve emotion regulation and lessen the severity of misophonia symptoms.

#### Addressing anger.

Anger is often linked to immediate, intense reactions to misophonic triggers and frequently emerges as a central feature, driving impulsive or avoidant behaviors [1,6,8,9,20,39–42,45]. In our network analysis, anger shows significant positive associations with anxiety, impulsivity, and nonacceptance, suggesting these elements may co-occur and intensify responses to triggers. Targeting anger is important not only for reducing distress but also for addressing interconnected processes such as anxiety, impulsivity, and nonacceptance, which contribute to the maintenance of misophonia severity.

Dialectical Behavior Therapy (DBT) provides strategies to manage anger by teaching individuals to pause and choose adaptive responses to triggers. Techniques such as the “STOP” skill and “Opposite Action” can help interrupt impulsive reactions driven by anger [26,98,99]. “STOP” encourages a mindful pause to observe emotions, reducing the immediate escalation of anger. “Opposite Action” involves taking action contrary to the emotional urge, such as engaging in calming activities instead of reacting aggressively, thereby weakening the anger response and reducing impulsive behaviors [100]. Cognitive restructuring can be adapted to manage misophonia-related anger to challenge and reframe thoughts that intensify anger, such as catastrophic beliefs about the intolerability of trigger sounds [91,101]. The UP has been shown to effectively reduce dysregulated anger across various clinical presentations [85,97,102]. Participants noted that recognizing unhelpful coping strategies and enhancing skills for managing emotional responses to trigger sounds were key to improving their emotional regulation [97]. Mindfulness-based interventions focus on cultivating nonjudgmental awareness of anger. By observing anger in the present moment without reacting, individuals can tolerate the initial emotional surge, reducing the likelihood of escalation [79–83,97]. These practices can help manage anger responses, leading to more adaptive coping strategies and potentially reducing the overall severity of misophonia symptoms.

#### Reducing anxiety and avoidance behaviors.

Interventions for anxiety in misophonia would benefit from addressing both anxiety reduction and avoidance behaviors. Anxiety in misophonia often manifests as anticipatory distress and avoidance of triggers, exacerbating the severity of symptoms. Cognitive-behavioral therapy (CBT) techniques, including cognitive restructuring, attentional shifting/task concentration, and emotion regulation/distress tolerance exercises, have shown promise in helping individuals reframe maladaptive thoughts and develop more adaptive coping strategies [84,91,101,103–105]. These techniques help patients reassess the intensity of discomfort tied to misophonic triggers, fostering tolerance and reducing reliance on avoidance behaviors. Building on this, the UP for misophonia specifically addresses distressing situations and challenges beliefs about discomfort, teaching patients to tolerate distress and manage their reactions to triggers [97]. By fostering increased tolerance of discomfort, patients gradually learn to manage their reactions to misophonic triggers by reassessing core beliefs that discomfort is unmanageable. This approach effectively reduces anxiety and avoidance behaviors, mitigating misophonia severity.

### Strengths and limitations

A key strength of this study is the use of network analysis to explore interrelations among misophonia severity, emotion regulation, anxiety, and anger. Unlike traditional methods, network analysis provides (1) a visual representation of how these factors interact, and (2) offers new insights into potential intervention targets [69].

The use of the Duke Misophonia Questionnaire (DMQ) is another strength, as it provides a comprehensive assessment of misophonia across multiple domains [62]. The DMQ includes subscales for Affective Responses, Physiological Responses, Cognitive Responses, and Impairment, allowing for a detailed evaluation of how misophonia affects individuals across various domains of functioning. Specifically, the impairment subscale of the DMQ, with its 12 items, offers a broader assessment of functional challenges compared to the MQ’s single-item measure. While the MQ’s concise approach efficiently captures an individual’s immediate perception of impairment, the DMQ’s more detailed items may uncover specific areas of life where misophonia has a notable impact. This difference in approach suggests that the two measures might be capturing slightly different dimensions of impairment. Our analysis revealed a significant difference between DMQ and MQ impairment scores, indicating that the MQ may capture a higher overall sense of impairment. This invites further exploration into how these tools can complement one another, offering different lenses through which researchers and clinicians might assess the condition’s full impact.

However, the study’s cross-sectional design limits the ability to infer causality, as the identified associations among emotion regulation, anxiety, anger, and misophonia severity do not establish causal pathways. Additionally, the robustness of network analysis depends on the variables included. While we focused on anxiety, anger, and emotion regulation based on their strong theoretical and empirical relevance to misophonia, other potentially relevant factors such as depressive symptoms were not included in this network model. This reflects both theoretical and methodological considerations: depressive symptoms, while commonly comorbid with misophonia, are not considered core or differentiating features of misophonia psychopathology, and including additional nodes would have increased model complexity and estimation demands, potentially compromising network stability [69,106]. Nonetheless, the omission of certain variables may have limited our ability to capture additional processes relevant to misophonia. For example, future studies incorporating depressive symptoms could help clarify whether shared or distinct pathways contribute to emotion regulation difficulties and misophonia severity. Similarly, other unmeasured confounds (e.g., sensory processing differences, comorbid psychiatric conditions, or environmental factors) may also have influenced the observed relationships. Nonetheless, our targeted approach allowed us to model key transdiagnostic processes most central to misophonia while preserving statistical stability and interpretability.

### Future directions

First, future research should prioritize longitudinal studies to clarify the directionality and causality of relationships among emotion regulation, anxiety, anger, and misophonia severity. These studies can determine to what extent deficits in emotion regulation, increased anxiety, or anger directly contribute to the onset and persistence of misophonia symptoms or if they are outcomes of the condition. Such insights can refine the timing and focus of interventions, ensuring they address the underlying mechanisms effectively.

Second, controlled clinical trials would be helpful in evaluating the efficacy of various therapeutic approaches. Current suggestions include targeting anxiety, anger, and nonacceptance of emotions using strategies adapted from third-wave cognitive-behavioral therapies like Acceptance and Commitment Therapy (ACT) and Dialectical Behavior Therapy (DBT), as well as the Unified Protocol (UP) and mindfulness-based interventions [107]. However, the evidence base for these interventions in treating misophonia specifically is still limited. Larger-scale trials are necessary to test these strategies and identify which components are most effective. Integrating a process-based therapeutic approach can also be valuable, allowing for a flexible application of techniques targeting specific biopsychosocial change processes most relevant to misophonia using evidence-based transdiagnostic treatment strategies, enabling more individualized and effective treatment plans.

Third, incorporating Ecological Momentary Assessment (EMA) in future research can significantly enhance the accuracy and depth of data, addressing the limitations of traditional retrospective self-report measures. EMA enables real-time data collection, capturing emotional reactions, coping strategies, and psychological change processes as they occur in daily life [108]. By providing a longitudinal, moment-to-moment examination of how emotions and behaviors fluctuate in response to misophonic triggers, EMA offers a more granular understanding of these dynamics over time. This method can help identify the processes most relevant to the maintenance or worsening of misophonia severity, informing the development of more precise and dynamic interventions. Additionally, integrating EMA into clinical trials can provide immediate feedback on intervention efficacy, supporting the refinement of individualized treatment plans and enhancing the assessment of long-term outcomes [109].

Fourth, future research should examine these processes in more demographically diverse and cross-cultural samples. The present sample was relatively homogeneous, limiting generalizability to broader populations. Expanding future samples to include greater variability in race, ethnicity, gender, socioeconomic status, and cultural context would allow for the examination of potential subgroup differences and strengthen the applicability of findings across diverse populations. Such efforts may also clarify whether the processes linking emotion regulation, anxiety, anger, and misophonia severity generalize across cultural frameworks and developmental backgrounds.

## Conclusion

This study contributes to the understanding of how emotion regulation, anxiety and anger relate to misophonia severity, highlighting potential areas for therapeutic focus. Our findings suggest that addressing anger, anxiety, and nonacceptance of emotions would be most beneficial in managing misophonia. Future research should explore these associations further, particularly through longitudinal studies and controlled trials, to elucidate causal pathways and refine interventions. Continued investigation of mechanisms underlying misophonia will inform the development of more precise and effective treatments tailored to the diverse presentations associated with this increasingly recognized condition.

## Supporting information

S1 File Network centrality and edge weight analyses. This file contains six supplemental figures providing detailed centrality and edge weight analyses from the network model. Included are figures depicting bridge strength centrality; bridge one-step and two-step expected influence; bootstrapped differences in strength centrality; bootstrapped differences in expected influence; bootstrapped differences in bridge strength centrality; and bootstrapped differences in edge weights.
(PDF)
