# Supplementary material for: Examining the role of emotion regulation, anger, and anxiety in misophonia: A network model
Source: PLoS One. 2025 Aug 18;20(8):e0329920. doi: 10.1371/journal.pone.0329920 (PMC12360532; doi:10.1371/journal.pone.0329920)

Supporting Information

Supplemental Fig 1. Bridge Strength Centrality. This figure displays the bridge strength centrality values for each node in the network. Bridge strength centrality measures the sum of absolute edge weights that connect nodes from different communities, indicating how central a node is in linking distinct sub-networks. In this network, “Clarity” and “Nonacceptance” show the highest bridge strength, suggesting that these nodes play a pivotal role in connecting the “Emotion Dysregulation and Misophonia” community to the “Emotional Clarity and Awareness” community.

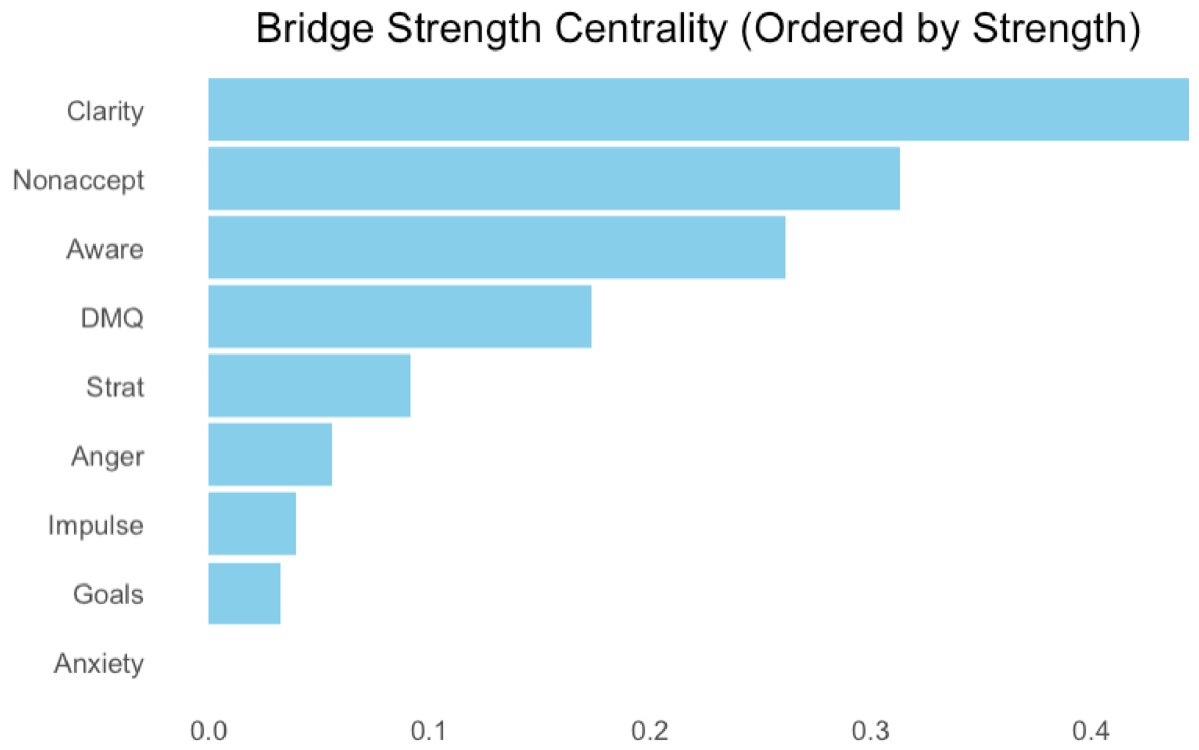

Supplementary Fig 2. Bridge One-Step and Two-Step Expected Influence. This figure illustrates the bridge one-step and two-step expected influence values for each node, organized by one-step expected influence. Bridge expected influence captures the sum of connections to other communities, either directly (one-step) or through neighboring nodes (two-step). Nodes like "Clarity" and "Nonacceptance" exhibit significant bridge one-step expected influence, indicating their direct role in connecting the "Emotion Dysregulation and Misophonia" and "Emotional Clarity and Awareness" communities. The two-step expected influence corroborates the importance of "Clarity" and "Nonacceptance" in connecting these two communities through neighboring nodes and provides further insight into the extended reach of nodes, showing how their influence propagates through intermediary connections to other parts of the network.

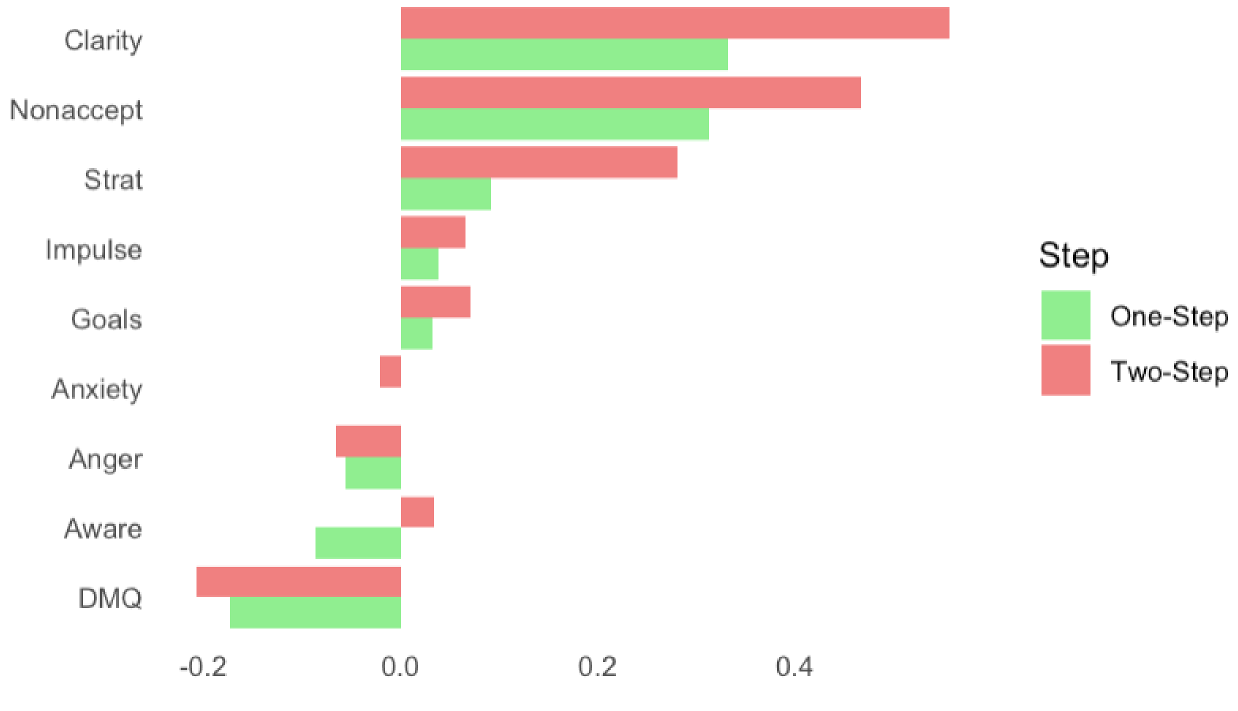

Supplemental Fig 3. Bootstrapped Differences in Strength Centrality. This heatmap presents the bootstrapped differences in strength centrality, which reflects the extent of a node's direct influence on others in the network. The nodes with the highest centrality values are "Strategies," "Nonacceptance," and "Impulsivity," as indicated by the darker shaded cells. The strength centrality values highlight which variables play pivotal roles in the network structure, and the bootstrapped differences help confirm the reliability of these findings across resamples. The black cells indicate significant differences in strength centrality, while the grey cells represent differences that are not statistically significant at the 0.05 level.

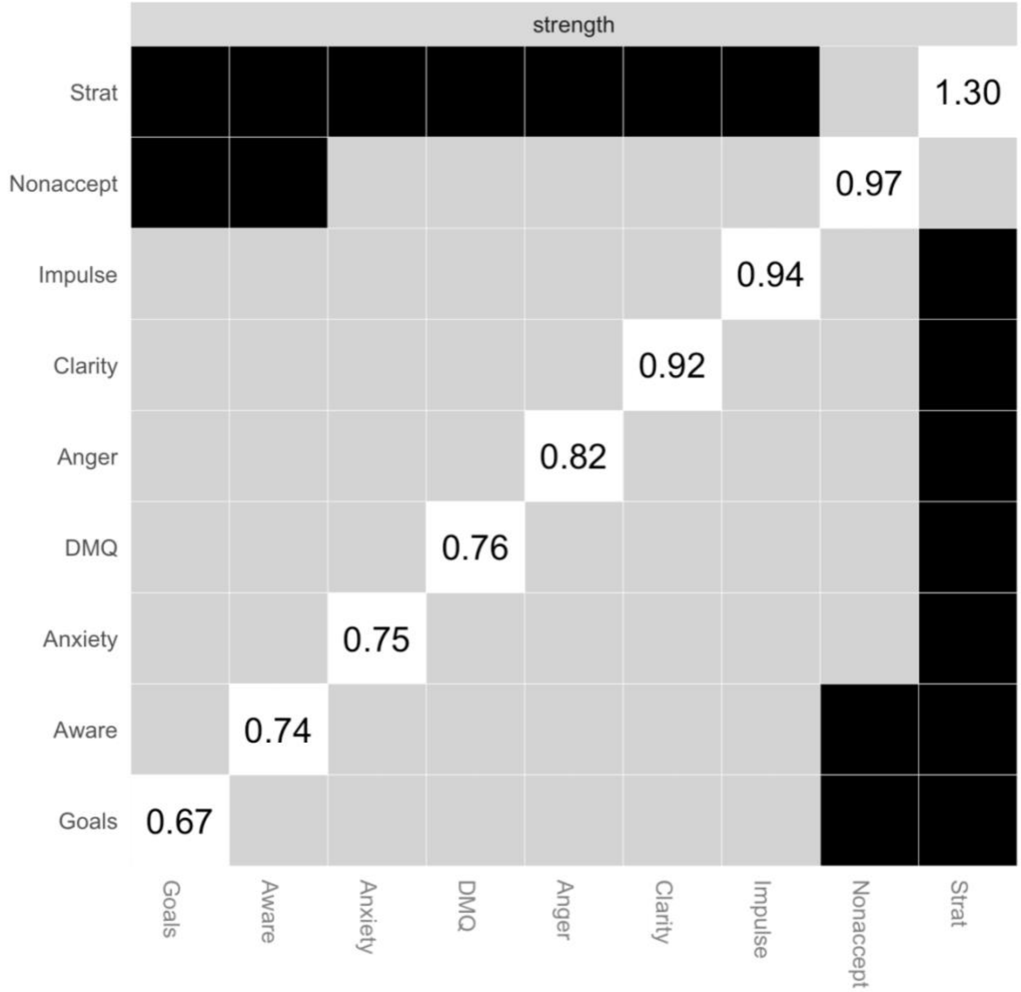

Supplemental Fig 4. Bootstrapped Differences in Expected Influence. This heatmap illustrates the bootstrapped differences in expected influence values, which capture both the direct and indirect influence of each node on others. The node "Strategies" has the highest expected influence, as shown by the darker shaded cell in the top row. Other key nodes with high expected influence include "Nonacceptance" and "Impulsivity." The expected influence values suggest which nodes have broader impacts within the network, and the differences reflect their stability across bootstrapped samples, providing confidence in these influential relationships. The black cells indicate significant differences in expected influence, while the grey cells represent differences that are not statistically significant at the 0.05 level.

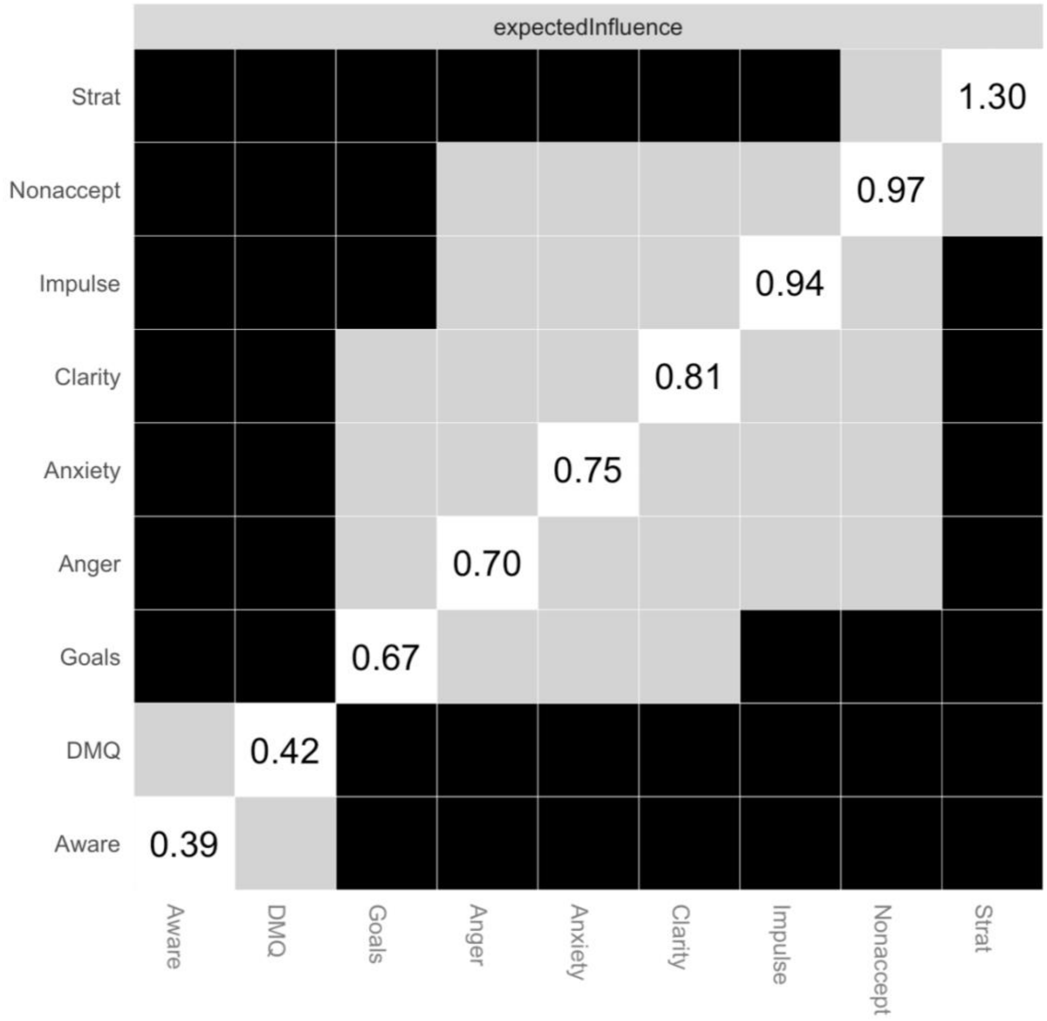

Supplemental Fig 5. Bootstrapped Differences in Bridge Strength Centrality. This heatmap shows the bootstrapped differences in bridge strength centrality values, indicating the extent to which each node serves as a connector between different communities in the network. The nodes "Clarity" and "Nonacceptance" have the highest bridge strength centralities, as shown by the higher values and darker shaded cells in their respective rows. These nodes play a crucial role in linking the "Emotion Dysregulation and Misophonia" and "Emotional Clarity and Awareness" communities, suggesting they act as key bridging points that facilitate interactions between these communities. The black cells indicate significant differences in bridge strength centrality, while the grey cells represent differences that are not statistically significant at the 0.05 level. The bootstrapped differences reflect the stability of these bridge centralities across resamples, providing confidence in the importance of these nodes in the overall network structure.

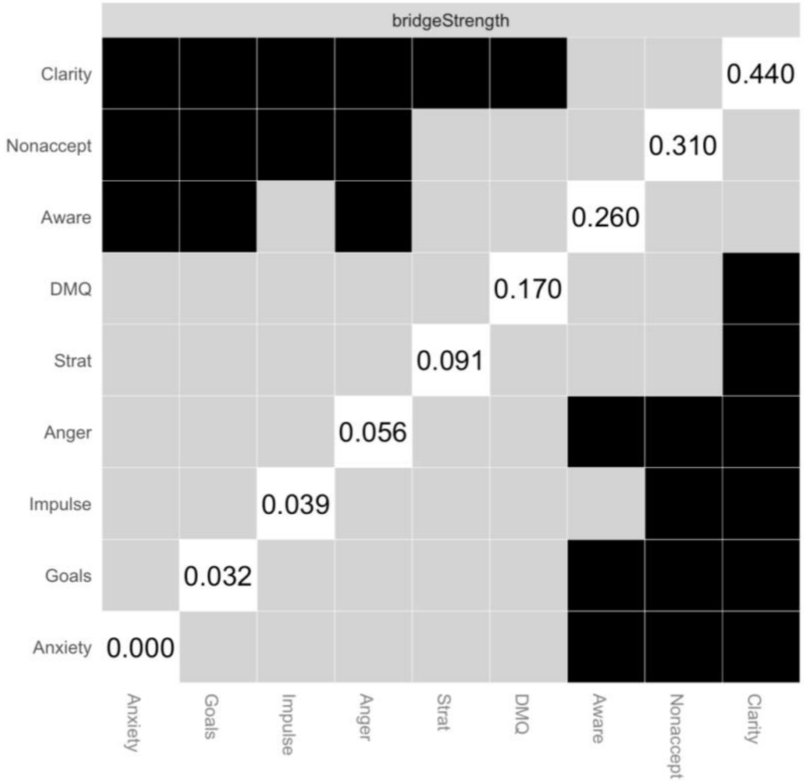

Supplemental Fig 6. Bootstrapped Differences in Edge Weights. This heatmap displays the bootstrapped differences in edge weights from the network. The edges represent partial correlations between pairs of nodes, with the color gradient indicating the magnitude of difference in the edge weights. The darker blue cells signify stronger positive associations, while the red cells indicate a negative association. The edges are organized from most positive associations (top right) to most negative (bottom left). These bootstrapped results provide insight into the variability of relationships between variables across different resamples, supporting the robustness of the network model. The black cells indicate significant differences in edge weights, while the grey cells represent differences that are not statistically significant at the 0.05 level.

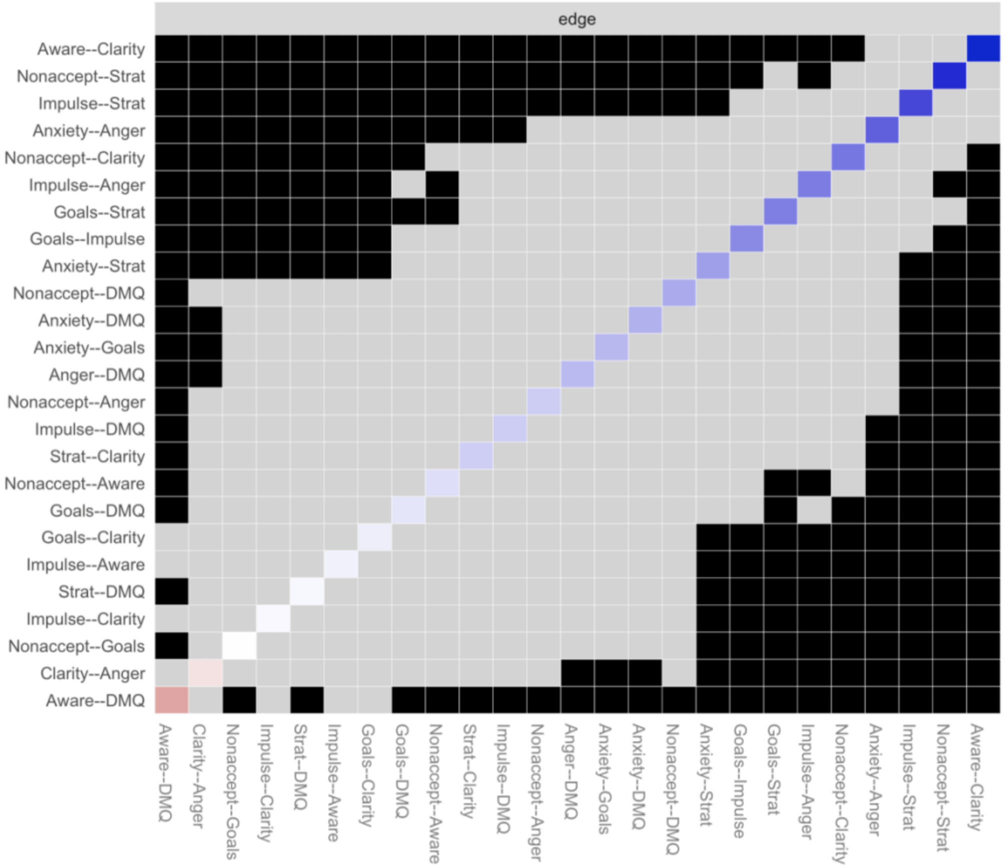

Supplement: S1 File — This file contains six supplemental figures providing detailed centrality and edge weight analyses from the network model. Included are figures depicting bridge strength centrality; bridge one-step and two-step expected influence; bootstrapped differences in strength centrality; bootstrapped differences in expected influence; bootstrapped differences in bridge strength centrality; and bootstrapped differences in edge weights. (PDF) [file pone.0329920.s001.pdf]
